# Indoor Mapping Guidance Algorithm of Rotary-Wing UAV Including Dead-End Situations

**DOI:** 10.3390/s19224854

**Published:** 2019-11-07

**Authors:** Jongho Park, Jaehyun Yoo

**Affiliations:** 1 Department of Military Digital Convergence, Ajou University, Suwon 16499, Korea; parkjo05@ajou.ac.kr; 2 Department of Electrical, Electronic and Control Engineering, Hankyong National University, Anseoung 17579, Korea

**Keywords:** unmanned aerial vehicle, mapping, indoor environment, guidance

## Abstract

A mapping guidance algorithm of a quadrotor for unknown indoor environments is proposed. A sensor with limited sensing range is assumed to be mounted on the quadrotor to obtain object data points. With obtained data, the quadrotor computes velocity vector and yaw commands to move around the object while maintaining a safe distance. The magnitude of the velocity vector is also controlled to prevent a collision. The distance transform method is applied to establish dead-end situation logic as well as exploration completion logic. When a dead-end situation occurs, the guidance algorithm of the quadrotor is switched to a particular maneuver. The proposed maneuver enables the quadrotor not only to escape from the dead-end situation, but also to find undiscovered area to continue mapping. Various numerical simulations are performed to verify the performance of the proposed mapping guidance algorithm.

## 1. Introduction

Research on Unmanned Aerial Vehicles (UAVs) has been conducted in recent years due to the various applications [1,2,3,4,5]. A high-fidelity UAV is able to deal with advanced missions including surveillance, reconnaissance, exploration, mapping, delivery, and disaster assistance in dangerous environments. Among various types of UAVs, a quadrotor has emerged as a popular platform because of the ability to perform vertical take-off and landing as well as the ability to quickly turn its direction. The quadrotor is a rotary-wing UAV composed of a cross configuration of two rods with four rotors [6,7,8,9,10]. Unlike a conventional rotary-wing UAV such as a helicopter [11], the quadrotor balances itself using the counter-rotating rotors. Therefore, a tail rotor is not required, which eventually leads to a reduced contact hazard. This is a great advantage with respect to collision avoidance if narrow space like indoor environment is considered.

Nowadays, three-dimensional data of indoor environment are needed in lots of applications such as navigation, mapping, surveillance, disaster management, and infrastructure inspection. In particular, it is extremely risky to send humans into hazardous indoor structure without prior knowledge of the considered environment. In this case, unmanned vehicles such as UAVs or mobile robots can be utilized to map and obtain three-dimensional data of the environment, instead of humans. Moreover, UAVs have an advantage of accessing disaster area with no constraints, whereas debris in such region can act as obstacles to mobile robots.

Indoor mapping has been investigated by several researchers. Arleo et al. [12] presented a map learning method using mobile robots in order to acquire a spatial-navigation model. A feed-forward neural network was used to interpret sensor reading. Luo and Lai [13] concentrated on multisensor fusion for an enriched indoor map construction. For consistent map construction, a modified particle swarm optimal technique was applied to remove the residual error caused by alignment of relative beacons. Khoshelham and Elberink [14] presented a mathematical model for obtaining three-dimensional object coordinates from raw image measurements using Kinect. Wen et al. [15] constructed a three-dimensional indoor mobile mapping system, with data fused by a two-dimensional laser scanner and RGB-D camera. Jung et al. [16] provided a method of solving indoor Simultaneous Localization And Mapping (SLAM) and relocation problems by exploiting ambient magnetic and radio sources. Jung et al. [17] formulated a feature-based SLAM technique incorporating a constrained least squares method. dos Santos et al. [18] focused on building a framework to map indoor spaces using an adaptive coarse-to-fine registration of RGB-D data obtained with a Kinect sensor. Lee et al. [19] suggested a system which efficiently captures indoor space’s features, and three-dimensional poses of a sensor were accurately estimated. Guerra et al. [20] described a modified approach to the monocular SLAM problem with data obtained from a human-deployed sensor. Li et al. [21] designed a framework for two-dimensional laser scan matching for the detection of point and line feature correspondences. Zhou et al. [22] developed a motion sensor-free approach and mobility analysis based on the sporadically collected crowd sourced Wi-Fi Received Signal Strength (RSS) data. The method focused on low time and labor cost in any unknown indoor environment. Jiang et al. [23] divided a scene map into eight areas and analyzed the probabilistic path vector of each area. Zhou et al. [24] applied a graph optimization method to construct an indoor map using pedestrian activities and context information. Tang et al. [25] proposed a vertex-to-edge weighted loop closure solution to minimize error in full RGB-D indoor SLAM. In addition, Qian et al. [26] presented an indoor SLAM algorithm via scan-to-map matching with a two-dimensional laser scanner and Inertial Measurement Unit (IMU).

The above studies mainly focused on a mapping system [12,14,18,19,21,22,23,24,25], sensor fusion [13,15,26], or solving a SLAM problem [16,17,20]. Furthermore, mobile robots were mostly considered in the above indoor mapping studies. However, a quarotor can be an appropriate platform for indoor mapping due to its properties mentioned earlier. On the other hand, a recently introduced RGB-D camera and three-dimensional laser scanner make it possible to provide data points of the surfaces of an object. To obtain full information of indoor environments without a collision, the quadrotor has to decide where to explore and what to avoid based on the object data points obtained during the flight. Therefore, development of an indoor mapping guidance algorithm of the quadrotor with obtained object data points is necessary. Meanwhile, a dead-end is a popular situation in indoor structures. In addition, without appropriate action, unmanned vehicle may be stuck in this situation, which possibly leads to the mission failure. Thus, indoor mapping guidance algorithm must consider this kind of situation, compared to the previous work [27].

In this study, a guidance algorithm for mapping indoor structure is proposed. The indoor environment considered in this study is restricted to a single floor structure. Thus, vertical movement between two floors or dealing with a ceiling or a stair is beyond the scope of this paper. In addition, collision with standing furniture such as a table or a chair is not considered. A quadrotor model with sensing device is considered as a platform [6,28]. It is assumed that the position and attitude of the quadrotor are known. That is, the dead reckoning issue of navigation system in indoor environments is beyond the scope of this paper. On the other hand, it is assumed that an object representing indoor environment is completely unknown to the quadrotor at initial state. The device with limited sensing range obtains three-dimensional data information from the object. In addition, a clustering method is conducted if multiple groups of data points are simultaneously detected. The obtained data points are utilized to produce a velocity vector command of the quadrotor. The velocity vector command helps the quadrotor move toward undiscovered area while maintaining a predefined distance with the object. A yaw command is used to being oriented toward the obtained data points so that the quadrotor can take care of a collision in the direction of the velocity vector. In addition, the magnitude of the velocity vector is controlled to avoid excessive maneuver of the quadrotor. A binary image is formed with the accumulated data points, and a distance transform method is applied to the binary image [29]. With the distance transform, the quadrotor determines whether the mapping exploration is completed or not. Furthermore, the distance transform image plane is used in dead-end situation logic. If a dead-end situation occurs, the guidance algorithm is immediately switched to the proposed dead-end phase maneuver to escape from the current situation. Numerical simulations are performed to demonstrate the performance of the proposed indoor mapping algorithm. A previous mapping guidance algorithm developed by Park and Kim [27] cannot handle dead-end situations, which may result in a collision. On the contrary, the simulation results show that the proposed guidance algorithm maps indoor environments without a collision, and dead-end situations are appropriately handled.

The contributions of this study are summarized as follows: first, a mapping guidance algorithm for an unknown indoor structure using a quadrotor is proposed. This method ensures both continuous mapping and collision avoidance. Second, a distance transform technique is introduced with obtained environment information to establish exploration completion logic and dead-end situation logic. Third, a particular dead-end phase maneuver is proposed for the quadrotor to escape from the dead-end situation and continue mapping undiscovered areas to complete the mission.

The paper is organized as follows: Section 2 provides dynamics, control, and data acquisition of a quadrotor system. In Section 3, the guidance algorithm for indoor mapping is proposed. Velocity vector command and yaw command are described. In addition, exploration completion logic and dead-end situation logic are proposed. In Section 4, simulation results are presented. Finally, the conclusions are given in Section 5.

## 2. Unmanned Aerial Vehicle System and Its Object Data Acquisition

### 2.1. Dynamics

**Figure 1:**
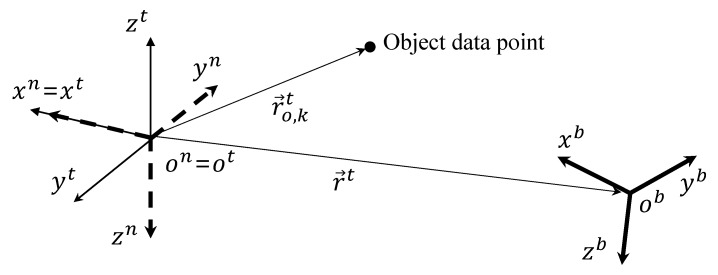
Coordinate systems.

In this study, a quadrotor is adopted as a rotary-wing UAV platform. Two frames are considered: an inertial frame $F_{i}$ and a body-fixed frame $F_{b}$, and coordinate systems are shown in Figure 1. A tangent–plane coordinate system is a geographic system whose axes ($x^{t}$, $y^{t}$, and $z^{t}$) are aligned with north, west, and up. A North-East-Down (NED) coordinate system is also a geographic system whose axes ($x^{n}$, $y^{n}$, and $z^{n}$) are aligned with north, east, and down. The tangent–plane and NED coordinate systems have a common origin ($o^{t}=o^{n}$) as shown in Figure 1. A body-fixed coordinate system is a coordinate system whose origin ($o^{b}$) is at the quadrotor’s center of mass and whose axes ($x^{b}$, $y^{b}$, and $z^{b}$) are aligned with the reference direction vector of the quadrotor. In Figure 1, ${\vec{r}}^{t}\equiv {\left[x^{t}\;y^{t}\;z^{t}\right]}^{T}$ is the position vector of the quadrotor in $F_{i}$, measured in the tangent–plane coordinate system, and ${\vec{r}}_{o,k}^{t}\equiv {\left[x_{o,k}^{t}\;y_{o,k}^{t}\;z_{o,k}^{t}\right]}^{T}$ is the position vector of *k*-th object data point in $F_{i}$, measured in the tangent–plane coordinate system. Note that a sensor that collects object data points is assumed to be located at the origin of the body-fixed coordinate system.

The quadrotor dynamics is governed by the following equations of motion [6]:

(1) $${\dot{\vec{r}}}^{t}=C_{t/n}C_{b/n}^{T}{\vec{v}}^{b},$$

(2) $${\dot{\vec{v}}}^{b}=\frac{1}{m}{\vec{F}}^{b}+C_{b/n}{\vec{g}}^{n}-{\vec{\omega }}^{b}\times {\vec{v}}^{b},$$

(3) $${\dot{\vec{\omega }}}^{b}=J^{-1}({\vec{M}}^{b}-{\vec{\omega }}^{b}\times J{\vec{\omega }}^{b}),$$

where ${\vec{v}}^{b}$ and ${\vec{\omega }}^{b}$ are the velocity of the quadrotor in $F_{b}$ and the angular velocity of $F_{b}$ with respect to $F_{i}$, measured in the body-fixed coordinate system, respectively, *m* and $J\equiv diag[J_{x},J_{y},J_{z}]$ are the mass and inertia matrix of the quadrotor, respectively, $C_{b/n}$ ($=C_{n/b}^{T}$) is the direction cosine matrix from the NED coordinate system to the body-fixed coordinate system, $C_{t/n}$ is the direction cosine matrix from the NED coordinate system to the tangent–plane coordinate system, ${\vec{F}}^{b}$ is the thrust force of the quadrotor measured in the body-fixed coordinate system, ${\vec{M}}^{b}$ is the thrust moment of the quadrotor measured in the body-fixed coordinate system, and ${\vec{g}}^{n}\equiv {\left[0\;0\;g\right]}^{T}$ is the gravity vector in $F_{i}$, measured in the NED coordinate system. Note that a Euler angle with a (1-2-3) sequence, $\vec{\Phi }\equiv {\left[\phi \;\theta \;\psi \right]}^{T}$, is defined between the NED and body-fixed coordinate systems.

### 2.2. Control Structure

As the quadrotor is an inherently under-actuated system, ϕ and θ are introduced as virtual control inputs to design the control system. In this study, a velocity controller is adopted, and therefore the velocity vector command, ${\vec{v}}_{c}^{t}\equiv {\left[v_{x,c}^{t}\;v_{y,c}^{t}\;v_{z,c}^{t}\right]}^{T}$, and the yaw command, $\psi_{c}$, are the inputs to the control system. In addition, these are generated by the proposed indoor mapping guidance algorithm. The final velocity command, ${\vec{v}}_{c}$, is refined as follows:

(4) $${\vec{v}}_{c}=v_{c}{\vec{v}}_{c}^{t}/{\left\|{\vec{v}}_{c}^{t}\right\|}_{2},$$

where $v_{c}$ is the magnitude of the velocity command.

A conventional nonlinear controller is used in this study. A feedback linearization technique is used to address the nonlinearity of the quadrotor’s dynamics. This method transforms the nonlinear equations, i.e., Equations (2) and (3), into the equivalent linear equations. To control the transformed linear system, a linear quadratic tracker is designed for the velocity control. The purpose of the linear quadratic tracker is to force the output to follow the desired output. Details of the feedback linearization and linear quadratic tracker techniques are addressed in [28] and [6], respectively.

### 2.3. Object Data Acquisition

**Figure 2:**
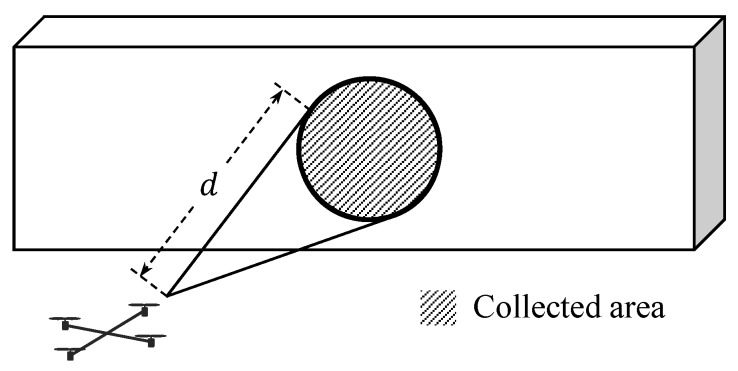
Acquisition of data points.

Figure 2 shows how the quadrotor acquires the object data points.

An object consists of a point cloud with $n_{t}$ data points that represent indoor environments. In addition, the quadrotor is equipped with a depth sensing device such as an RGB-D camera, which enables the UAV to achieve three-dimensional object data information. If ${\vec{r}}_{o,k}^{t}$ in Figure 1 is within sensing range, *d*, of the device, then ${\vec{r}}_{o,k}^{t}$ is collected by the quadrotor as follows:

(5) $$\left\{\begin{array}{cc} \mathrm{Object}\;\mathrm{data}\;\mathrm{point}\;\mathrm{collected},\; & \mathrm{if}\;\|{\vec{r}}_{o,k}^{t}-{\vec{r}}^{t}{\|}_{2}\leq d,\\ \mathrm{Not}\;\mathrm{collected}, & \mathrm{otherwise} \end{array}\right.$$

Therefore, at a certain time, only a small part of the object may be sensed by the quadrotor as shown in Figure 2. However, as time goes on, the quadrotor accumulates the obtained point cloud of the object to reconstruct indoor environments.

## 3. Indoor Mapping Guidance Algorithm

### 3.1. Velocity Vector and Yaw Commands

Let us assume that $n_{c}$ data points are sensed by the quadrotor. Once the object data points are collected, the quadrotor maps the object while following the path to move around the detected data points. A certain point representing the detected data points should be extracted, and the one with the minimum distance with respect to the quadrotor is selected in this study:

(6) $${\vec{r}}_{o,min}^{t}=\underset{k}{\mathrm{argmin}}\left\{\|{\vec{r}}_{o,k}^{t}-{\vec{r}}^{t}{\|}_{2}\;|\;\;k\in \left\{1,\cdots ,n_{c}\right\}\right\}.$$

Let us define ${\vec{r}}^{t,xy}$ and ${\vec{r}}_{o,min}^{t,xy}$ as the (x,y)-component vector of ${\vec{r}}^{t}$ and ${\vec{r}}_{o,min}^{t}$, respectively, in the tangent–plane coordinate system. Then, ${\vec{l}}_{t}$ can be defined as a vector perpendicular to $\left({\vec{r}}^{t,xy}-{\vec{r}}_{o,min}^{t,xy}\right)$, which can be obtained by computing the null space of ${\left({\vec{r}}^{t,xy}-{\vec{r}}_{o,min}^{t,xy}\right)}^{T}$. Finally, a vector ${\vec{v}}_{t}^{t,xy}$ can be obtained as follows:

(7) $${\vec{v}}_{t}^{t,xy}={\vec{l}}_{t}/{\left\|{\vec{l}}_{t}\right\|}_{2}.$$

The vector ${\vec{v}}_{t}^{t,xy}$ is used to move the quadrotor around the object for mapping. On the other hand, ${\vec{l}}_{n}$ can be defined as follows:

(8) $${\vec{l}}_{n}={\vec{r}}^{t,xy}-{\vec{r}}_{o,min}^{t,xy}.$$

Then, a vector ${\vec{v}}_{n}^{t,xy}$ can be obtained as follows:

(9) $${\vec{v}}_{n}^{t,xy}={\vec{l}}_{n}/{\left\|{\vec{l}}_{n}\right\|}_{2}.$$

The vector ${\vec{v}}_{n}^{t,xy}$ is used to maintain a safe distance with the object during the mapping. The distance between ${\vec{r}}^{t,xy}$ and ${\vec{r}}_{o,min}^{t,xy}$ is defined as $d_{min}$. In addition, an ideal distance, $\ell_{id}$, between the quadrotor and the object is introduced. If $d_{min}$ is smaller than $\ell_{id}$, then the quadrotor should move away from the object to maintain a safe distance. On the other hand, if $d_{min}$ is larger than $\ell_{id}$, then the quadrotor should move toward the object so that the quadrotor can map the object from the ideal distance.

A weighting parameter, $c_{n}$, is introduced to generate the velocity command $v_{x,c}^{t}$ and $v_{y,c}^{t}$ by combining ${\vec{v}}_{t}^{t,xy}$ and ${\vec{v}}_{n}^{t,xy}$ as follows:

(10) $${\left[\begin{array}{cc} v_{x,c}^{t} & v_{y,c}^{t} \end{array}\right]}^{T}=\left(1-|c_{n}|\right){\vec{v}}_{t}^{t,xy}+c_{n}{\vec{v}}_{n}^{t,xy},$$

where

(11) $$\left\{\begin{array}{cc} c_{n}=-c_{bf}\tilde{\ell },\;\\ c_{n}=1 & \mathrm{if}\;-c_{bf}\tilde{\ell }>1,\\ c_{n}=-1 & \mathrm{if}\;-c_{bf}\tilde{\ell }<-1, \end{array}\right.$$

with $\tilde{\ell }=d_{min}-\ell_{id}$. Note that $c_{bf}>0$ is a design parameter. On the other hand, $v_{z,c}^{t}$ is used to maintain the altitude of the quadrotor as follows:

(12) $$v_{z,c}^{t}=c_{z}\left(z_{0}^{t}-z^{t}\right),$$

where $z_{0}^{t}$ is the initial z-component of the quadrotor in the tangent–plane coordinate system, and $c_{z}$ is a design parameter.

The yaw command, $\psi_{c}$, is used to orient $+x^{b}$-axis of the body-fixed coordinate system toward the obtained object data points as follows:

(13) $$\psi_{c}=\arctan\frac{y_{o,min}^{n}-y^{n}}{x_{o,min}^{n}-x^{n}},$$

where ${\vec{r}}^{n}\equiv {\left[x^{n}\;y^{n}\;z^{n}\right]}^{T}$ is the position vector of the quadrotor in $F_{i}$, measured in the NED coordinate system, and ${\left[x_{o,min}^{n}\;y_{o,min}^{n}\;z_{o,min}^{n}\right]}^{T}$ can be obtained as follows:

(14) $${\left[x_{o,min}^{n}\;y_{o,min}^{n}\;z_{o,min}^{n}\right]}^{T}=C_{t/n}^{T}{\vec{r}}_{o,min}^{t}.$$

As Equations (10) and (14) are considered, the quadrotor may move along $\pm y^{b}$-axis of the body-fixed coordinate system for mapping (direction of ${\vec{v}}_{t}^{t,xy}$). Among two directions, $+y^{b}$-axis is chosen in this study. As the null space computation can produce two different vectors of ${\vec{l}}_{t}$, the following equation should be considered before conducting Equation (7):

(15) $${\vec{l}}_{t}=-{\vec{l}}_{t}\;\;\;\mathrm{if}\;{\vec{l}}_{t}\cdot {\vec{e}}_{y}^{t,xy},$$

where ${\vec{e}}_{y}^{t,xy}$ is the (x,y)-component vector of ${\vec{e}}_{y}^{t}$, and ${\vec{e}}_{y}^{t}$ can be obtained as follows:

(16) $${\vec{e}}_{y}^{t}=C_{t/n}C_{b/n}^{T}{\left[0\;1\;0\right]}^{T}.$$

### 3.2. Velocity Magnitude Command

**Figure 3:**
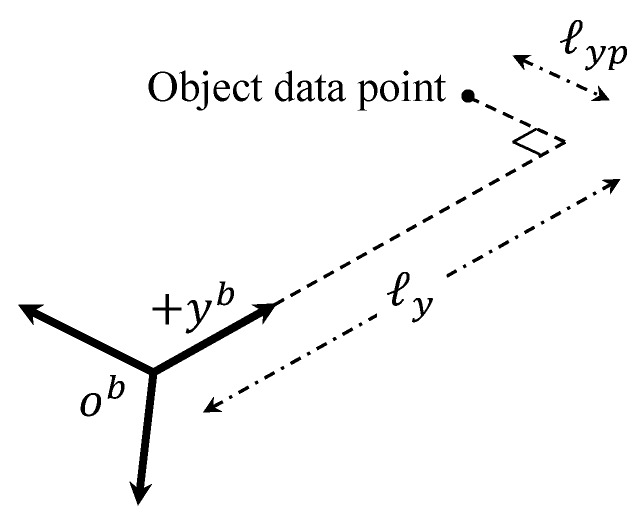
Object data point in the direction of $+y_{b}$-axis.

The magnitude of the velocity command, $v_{c}$, is adjusted to prevent a collision that may occur along the $+y_{b}$-axis of the quadrotor. Let us define $\ell_{yp}$ as the minimum distance between the $+y_{b}$-axis and the obtained object data points, as shown in Figure 3.

If $\ell_{yp}$ is smaller than a predefined threshold, $\ell_{yd}$, then the distance from the quadrotor to the considered data point along $+y_{b}$-axis as shown in Figure 3 can be calculated as follows:

(17) $$\ell_{y}=\left({\vec{r}}_{o,k}^{t}-{\vec{r}}^{t}\right)\cdot {\vec{e}}_{y}^{t}.$$

If $\ell_{y}$ is smaller than $\ell_{y_{max}}$, then an object along the $+y_{b}$-axis may be a threat to the quadrotor. In this case, the quadrotor reduces the magnitude of the velocity until $\ell_{y}$ reaches minimum allowable magnitude of the velocity, $v_{min}$, at $\ell_{y}=\ell_{y_{min}}$ with the following equation:

(18) $$\left\{\begin{array}{cc} v_{c}=v_{0},\; & \mathrm{if}\;\ell_{y_{\max}}\leq \ell_{y},\\ v_{c}=\frac{v_{0}-v_{min}}{\ell_{y_{max}}-\ell_{y_{min}}}\left(\ell_{y}-\ell_{y_{min}}\right)+v_{min}, & \mathrm{if}\;\ell_{y_{\min}}\leq \ell_{y}<\ell_{y_{\max}},\\ v_{c}=v_{min}, & \mathrm{otherwise}, \end{array}\right.$$

where $v_{0}$ is the normal magnitude of the velocity during the mapping. As this maneuver continues, ${\vec{r}}_{o,min}^{t}$ is eventually selected from the object surface along the $+y_{b}$-axis of the quadrotor. This means that the quadrotor turns its $+x_{b}$-axis toward the new side of the object using Equation (13). Then, the quadrotor continues mapping the object using the velocity command of Equation (10).

### 3.3. Exploration Completion Logic

**Figure 4:**
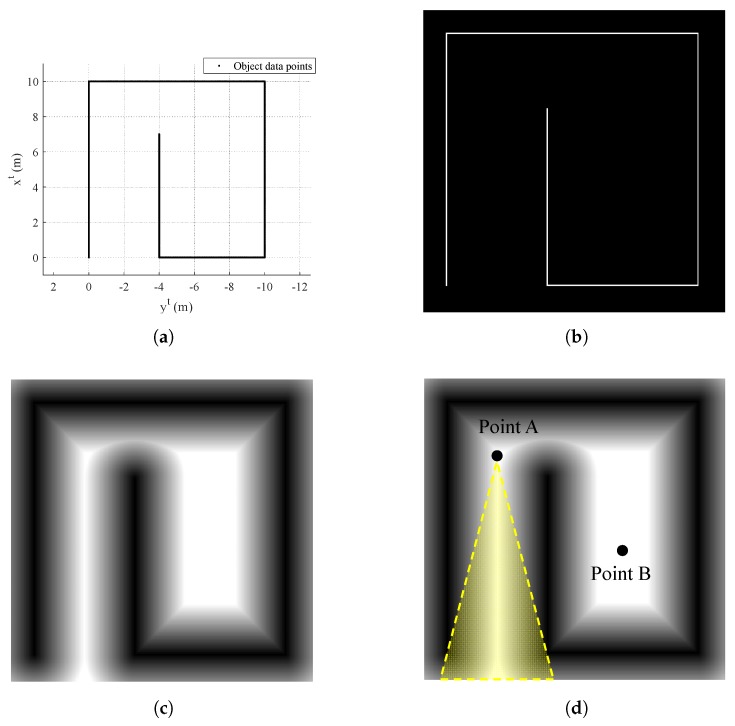
Image processing for exploration completion logic: (**a**) obtained object data points (top view, tangent–plane coordinate system); (**b**) binary image; (**c**) distance transform; and (**d**) two different UAV locations.

During the mapping process, the quadrotor needs to know whether the current area (or the past area) has a remaining part of the environment to be mapped or not. This can be determined by examining the Euclidean distance transform of the binary image that is formed by collected object data points. Figure 4 shows the sequence how to obtain the image plane with distance transform applied.

Let us assume that points in Figure 4a are collected by the quadrotor. Then, binary image as shown in Figure 4b can be obtained where object is represented by one (white pixels in Figure 4b) and empty space is represented by zero (black pixels in Figure 4b). Note that discretization should be conducted because the image plane consists of pixels. Now, distance transform is applied to the binary image plane as shown in Figure 4c.

With this distance transform, let us assume that the quadrotor is at point A as shown in Figure 4d. At point A, the quadrotor is partially surrounded by zero due to the yellow-shaded region as shown in Figure 4d. In other words, there is yet an undiscovered part of the environment from the view at point A. On the other hand, if the quadrotor is located at point B in Figure 4d, the quadrotor is perfectly surrounded by zero. If any straight line spreads out starting from point B at any direction, the line eventually meets the pixel with zero value. Note that the line only passes through the discovered area. That is, if at least one line meets the pixel which is not discovered in the past, then the line stops at the pixel and is unable to meet the object. This examination is repeated for every position of the UAV in the past history with currently updated data points. If every trajectory of the UAV is surrounded by zero value, then the exploration is completed.

This strategy is possible because only indoor mapping is considered in this study. Namely, as the UAV starts the mapping process inside the environment, every part of the trajectory will ultimately be surrounded by the environment.

### 3.4. Dead-End Situation Logic

**Figure 5:**
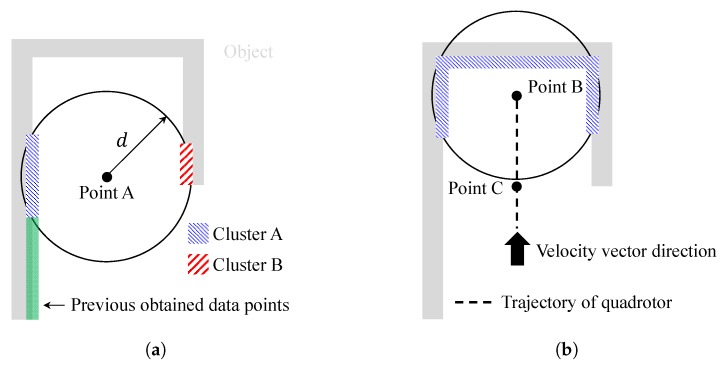
Narrow aisle example: (**a**) clustering; and (**b**) dead-end situation.

The proposed exploration completion logic using distance transform also works well for dead-end situations. The narrow aisle is a typical dead-end situation as shown in Figure 5. Furthermore, in narrow aisles, object data points can be simultaneously collected in multiple groups as shown in Figure 5a where the quadrotor is located at point A. To deal with this problem, clustering is applied. For example, there are two clusters in Figure 5a. Among these two clusters, cluster A is recognized as an extension of previously obtained object data points. Therefore, it is reasonable to choose only cluster A’s data points in producing velocity vector and yaw commands of Equations (10) and (13).

Now, let us assume that the quadrotor is located at point B in Figure 5b, as the quadrotor continues mapping. The UAV can recognize that it is in a dead-end situation by performing image processing in Figure 4. Let us denote the guidance algorithm of Equations (10), (13), and Equation (18) as a normal maneuver. If every straight line starting from the current position (point B in Figure 5b) which heads toward the quadrotor’s velocity vector direction meets the pixel with zero value of a distance transform image plane, then the quadrotor switches the guidance law from the normal maneuver to a dead-end phase I maneuver. When the quadrotor enters the dead-end phase I maneuver, the latest position is calculated where at least one straight line does not meet the pixel with zero value. Let us denote this position as point C, which is shown in Figure 5b. At point C, the quadrotor can continue mapping with an undiscovered area. Therefore, during the dead-end phase I maneuver, the quadrotor moves from point B to point C. When the quadrotor nearly reaches point C, the magnitude of the velocity reduces to $v_{min}$ so that the guidance law can be changed smoothly. Note that multiple clusters in Figure 5a merge into a single cluster in Figure 5b as a dead-end situation occurs.

When the quadrotor passes point C, the guidance law is switched to dead-end phase II maneuver. As the quadrotor enters the dead-end phase II maneuver, the velocity vector command is adjusted to the direction which does not meet the pixel with zero value of distance transform image plane. This direction is already calculated during the dead-end phase I maneuver. During the dead-end phase II maneuver, the magnitude of the velocity is set to $v_{min}$ to avoid excessive maneuvers of the quadrotor. After $t_{m3}$ seconds, the quadrotor switches the guidance algorithm to normal maneuver to continue mapping. Note that $t_{m3}$ is a design parameter.

During dead-end phase I and phase II maneuvers, a collision may occur with previously obtained object data points. Therefore, an additional guidance algorithm similar to ${\vec{v}}_{n}^{t,xy}$ in Equation (10) must be considered if the object is blocking the velocity vector of the quadrotor. This process is repeated until the mapping exploration is completed. The proposed strategy is summarized in Algorithm 1. In Algorithm 1, the quadrotor continues mapping when Explr is set as 1, whereas Explr=0 means that the mapping is completed. In addition, MM is the current maneuver of the quadrotor where 1, 2, and 3 represent normal maneuver, dead-end phase I maneuver, and dead-end phase II maneuver, respectively. Finally, M1to2 and M3to1 are switching maneuver flags.
**Algorithm 1:** Overall strategy including dead-end situation.
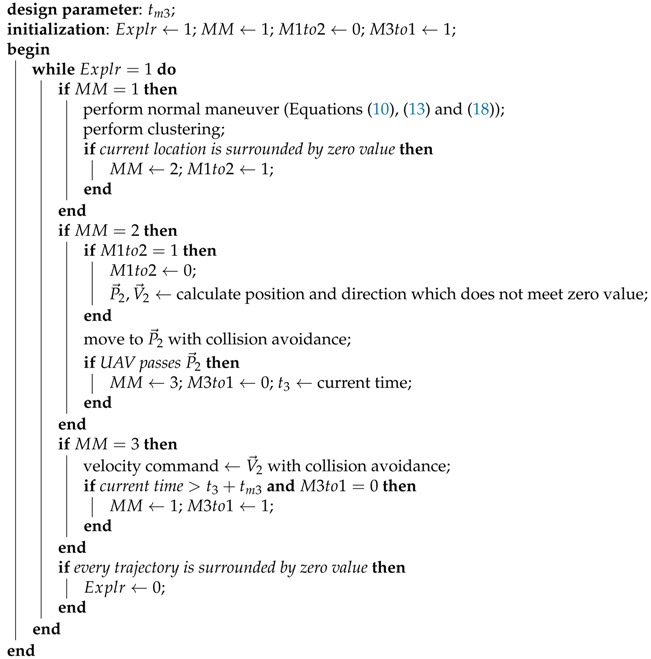


## 4. Numerical Simulation

Various numerical simulations are conducted to demonstrate the performance of the proposed guidance algorithm. The parameters of the quadrotor and mapping used in the numerical simulation are summarized in Table 1.

The sampling time of object data acquisition is set as 0.2 s. This is because vision systems commonly require large computational effort in practical application. This means that the proposed guidance law is updated every 0.2 s and the quadrotor’s control system uses the latest updated command.

### 4.1. Simulation I: Single Room

In Simulation I, the single room is considered as an indoor environment. The object consists of a point cloud with 6601 data points, which represent its external surface. The initial position of the quadrotor is set as ${\left[3\;-2\;\;2\right]}^{T}$ in the tangent–plane coordinate system, and the initial yaw angle is set as $0^{\circ }$.

**Figure 6:**
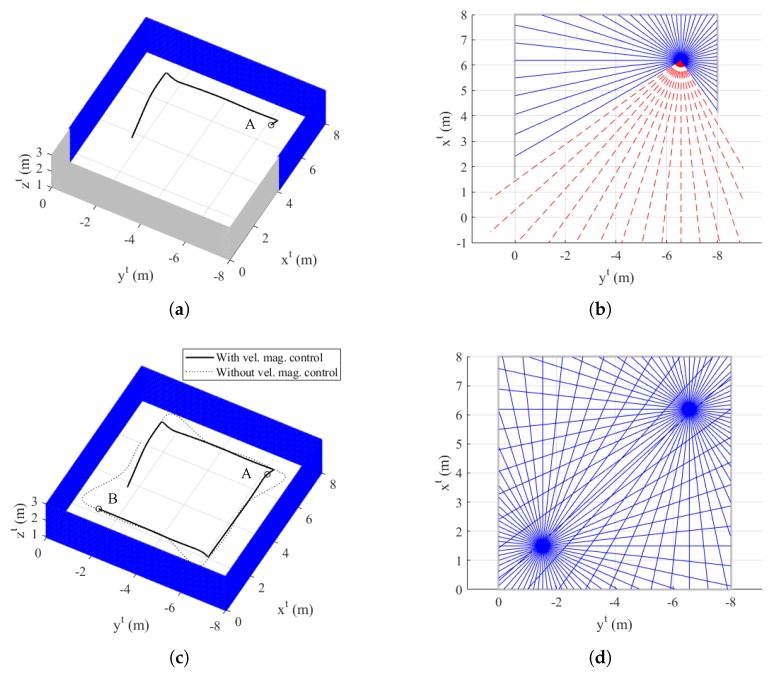
Simulation I result: (**a**) trajectory of the quadrotor and mapping at t=16.0 s (azimuth: −62${}^{\circ }$ and elevation: 50${}^{\circ }$, tangent–plane coordinate system); (**b**) exploration completion check at t=16.0 s; (**c**) trajectory of the quadrotor and mapping at t=32.0 s (azimuth: −62${}^{\circ }$ and elevation: 50${}^{\circ }$, tangent–plane coordinate system); and (**d**) exploration completion check at t=32.0 s.

**Figure 7:**
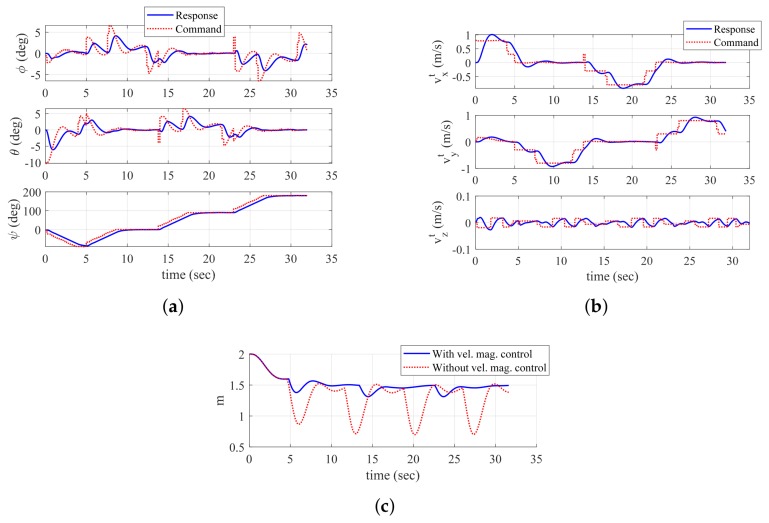
Time histories of UAV-related parameters (Simulation I): (**a**) attitude; (**b**) velocity (tangent–plane coordinate system); and (**c**) minimum distance between UAV and object.

Figure 6 shows the trajectory of the quadrotor, mapping result, and exploration completion examination at t=16.0 s and t=32.0 s. In addition, Figure 7 shows the time responses of the attitude and velocity of the quadrotor, and the minimum distance between the quadrotor and the object. In Figure 6a,c, the gray-shaded area represents the object, and the blue-shaded area represents the mapping result (collected data points) obtained by the sensing device. In addition, in Figure 6c, the solid line shows the trajectory with velocity magnitude command of Equation (18), whereas the dotted line shows the trajectory without a velocity magnitude command. Note from Figure 7c that the distance between the quadrotor and the object reaches the minimum distance 0.6980 m when Equation (18) is not applied. However, with Equation (18), the minimum distance is 1.3117 m, which shows a significant improvement considering that $\ell_{id}$ is set as 1.5 m.

At t=16.0 s, the quadrotor is located at point A as shown in Figure 6a. In addition, Figure 6b shows the result of the proposed exploration completion examination at this point. Straight lines starting from point A are expressed in Figure 6b. Blue-solid lines meet the pixel with zero value of the distance transform image plane, whereas red-dashed lines do not meet the object. Therefore, the quadrotor perceives that there is undiscovered area, and continues mapping. At t=32.0 s, the quadrotor is located at point B as shown in Figure 6c. In addition, Figure 6d shows the result of the exploration completion examination at this point. As shown in Figure 6d, every line from point B is bounded by the object. Furthermore, every line from point A at t=32.0 s is also bounded. This means that the exploration completion examination result of the past trajectory can be changed by currently updated data points. As every position of the quadrotor satisfies the proposed exploration completion logic, the mapping process is finally completed.

Dotted lines in Figure 7a,b represent the generated control commands, and solid lines represent the actual responses. As shown in Figure 7a, yaw angle changes whenever the quadrotor discovers a new surface of the environment. Thanks to Equation (18), the quarotor can decrease the magnitude of the velocity before changing the yaw angle as shown in Figure 7b.

### 4.2. Simulation II: L-Shaped Aisle

In Simulation II, the L-shaped aisle is considered to deal with dead-end situations. The object consists of a point cloud with 6762 data points. The initial position of the quadrotor is set as ${\left[0\;0\;2\right]}^{T}$ in the tangent–plane coordinate system, and the initial yaw angle is set as $180^{\circ }$. In this simulation, the proposed guidance algorithm is compared to the previous algorithm [27].

**Figure 8:**
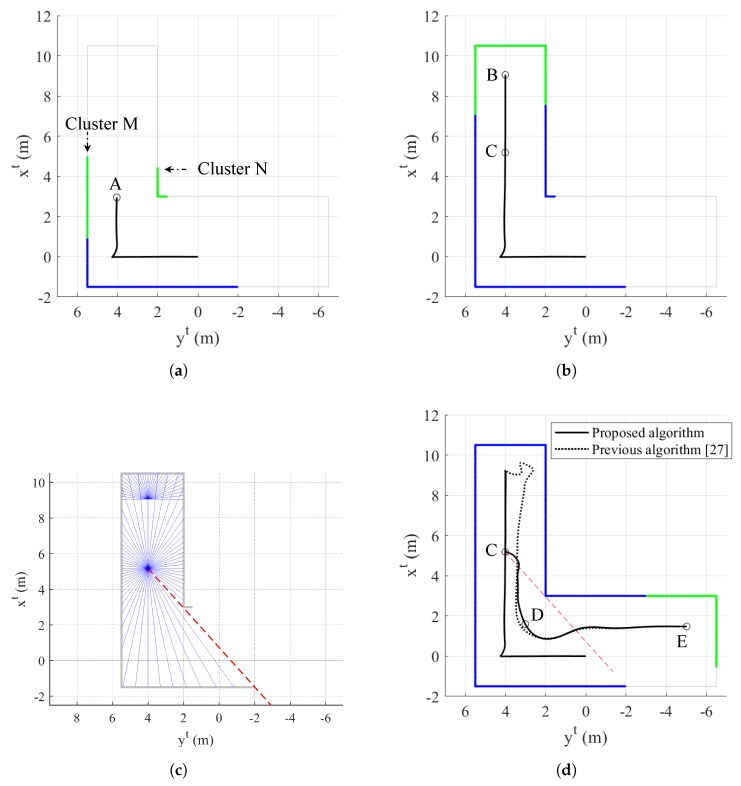
Simulation II result: (**a**) trajectory of the quadrotor and mapping at t=12.0 s (top view, tangent–plane coordinate system); (**b**) trajectory of the quadrotor and mapping at t=19.8 s (top view, tangent–plane coordinate system); (**c**) exploration completion check at t=19.8 s; and (**d**) trajectory of the quadrotor and mapping at t=47.0 s (top view, tangent–plane coordinate system).

**Figure 9:**
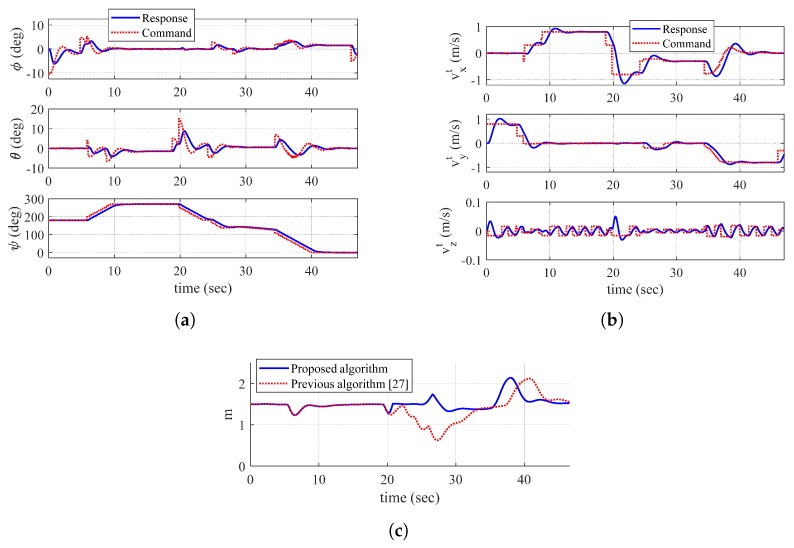
Time histories of UAV-related parameters (Simulation II): (**a**) attitude; (**b**) velocity (tangent–plane coordinate system); and (**c**) minimum distance between UAV and object.

Figure 8 shows the trajectory of the quadrotor and mapping result at t=12.0 s, t=19.8 s, and t=47.0 s, and exploration completion examination at t=19.8 s. In addition, Figure 9 shows the time responses of the attitude and velocity of the quadrotor, and the minimum distance between the quadrotor and the object. In Figure 8a,b,d, the blue-shaded area represents total collected object data points and the green-shaded area represents data points obtained at current time step. In Figure 8d and Figure 9c, the solid line shows the trajectory of the quadrotor and the minimum distance produced by the proposed algorithm, respectively, whereas the dotted line shows the trajectory and the minimum distance produced by the previous algorithm, respectively.

At t=12.0 s in Figure 8a, the quadrotor is located at point A and encounters a new side of the object. The proposed algorithm conducts the clustering process, which results in producing two clusters, denoted as M and N, in Figure 8a. As cluster M consists of the points which are connected to previously obtained data points, the quadrotor utilizes the points in cluster M to generate the velocity vector and yaw commands. Therefore, regardless of the points in cluster N, the quadrotor maintains the safe distance with the data points in cluster M as shown in Figure 8b. When the quadrotor reaches point B at t=19.8 s, straight lines starting from point B (current position) meet the pixel with zero value of distance transform image plane as shown in Figure 8c. Note that only half range ($180^{\circ }$) which includes the direction of the velocity vector is examined.

Now, the guidance algorithm is switched to the dead-end phase I maneuver and the quadrotor finds the latest point which is not entirely surrounded by zero value of distance transform image plane. This point is marked as point C in Figure 8b, and there is a red-dashed line starting from point C which does not meet any collected data points at current time step as shown in Figure 8c. This means that undiscovered area can be found if the quadrotor heads in this direction. With the dead-end phase I maneuver, the quadrotor moves back to point C and avoids a collision with dead-end object.

At t=25.4 s, the quadrotor passes point C and the guidance algorithm is switched to the dead-end phase II maneuver as shown in Figure 8d. The velocity vector command should be adjusted to the direction which is shown as red-dashed line in Figure 8d. At first, the quadrotor tracks the red-dashed line. However, as soon as the distance between the quadrotor and the obtained data point becomes $\ell_{id}$, the quadrotor deviates from the red-dashed line to prevent a collision. Now, the quadrotor maintains the distance $\ell_{id}$ from the object while trying to follow the red-dashed line.

After $t_{m3}$ seconds, the quadrotor is located at point D in Figure 8d, and the guidance algorithm is switched to the normal maneuver. It is shown that the quarotor successfully manages to escape from dead-end situation and finds undiscovered area with the proposed algorithm. Then, the quadrotor continues mapping with the normal maneuver as shown in Figure 8d.

As shown in Figure 8d, the previous guidance algorithm does not properly handle dead-end situations. In addition, the distance between the quadrotor and the object reaches the minimum distance 0.6244 m as shown in Figure 9c, which may lead to a collision if the volume of the quadrotor is considered. On the other hand, it is shown that the proposed guidance algorithm appropriately handles dead-end situation where the minimum distance is 1.2335 m.

### 4.3. Simulation III: Complicated Indoor Environment

In Simulation III, complicated environment is considered to verify the overall performance of the proposed guidance algorithm. The object consists of a point cloud with 42,354 data points. The initial position of the quadrotor is set as ${\left[2\;15\;2\right]}^{T}$ in the tangent–plane coordinate system, and the initial yaw angle is set as $180^{\circ }$.

**Figure 10:**
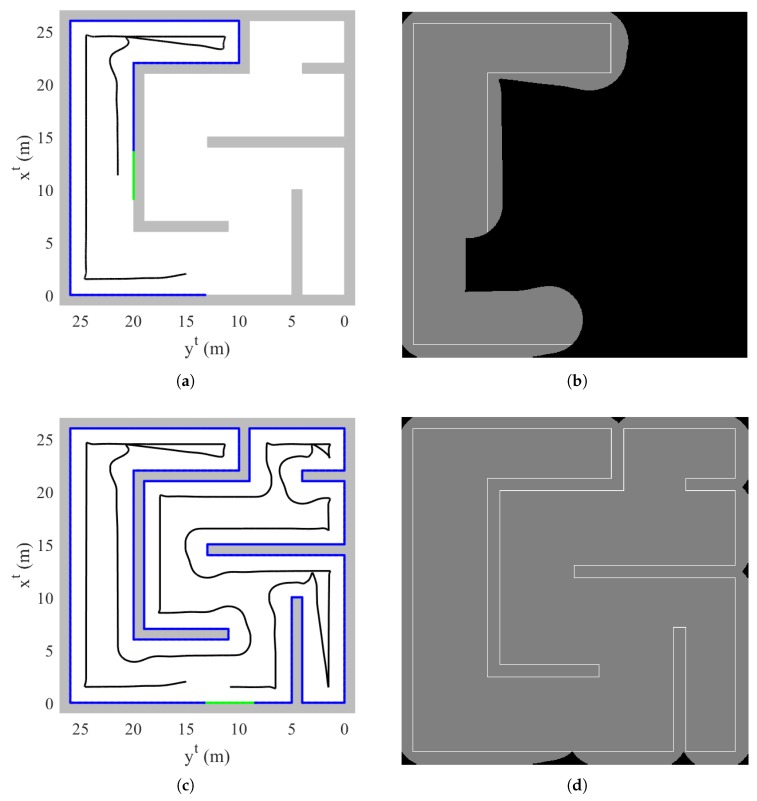
Simulation III result: (**a**) trajectory of the quadrotor and mapping at t=98.0 s (top view, tangent–plane coordinate system); (**b**) discovered area at t=98.0 s; (**c**) trajectory of the quadrotor and mapping at t=318.4 s (top view, tangent–plane coordinate system); (**b**) discovered area at t=318.4 s.

**Figure 11:**
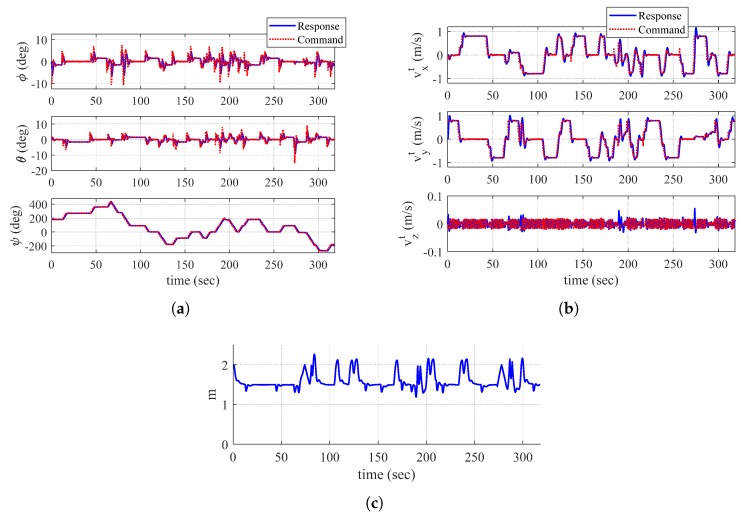
Time histories of UAV-related parameters (Simulation III): (**a**) attitude; (**b**) velocity (tangent–plane coordinate system); and (**c**) minimum distance between UAV and object.

The total flight time is 318.4 s. Figure 10 shows the trajectory of the quadrotor, mapping result, and discovered area at t=98.0 s and t=318.4 s. In addition, Figure 11 shows the time responses of the attitude and velocity of the quadrotor, and the minimum distance between the quadrotor and the object. In Figure 10b,d, the gray-shaded area represents the discovered area at a current time step, whereas the black-shaded area represents the undiscovered area. In addition, the white line shows the obtained obstacle data points.

At t=98.0 s with currently obtained data points, the quadorotor is perfectly surrounded by zero value of distance transform image plane as shown in Figure 10a. However, the quadrotor does not switch the guidance algorithm to a dead-end phase I maneuver because there is an undiscovered area in the direction of the velocity vector as shown in Figure 10b. When conducting the proposed dead-end situation logic, a straight line cannot pass through an undiscovered area. Therefore, the line is unable to meet a zero value of distance transform, which results in continuing the normal maneuver.

At t=318.4 s, as every trajectory of the quadrotor is surrounded by the obtained data points, the quadrotor determines that the exploration is completed. Throughout the mapping, as shown in Figure 10c, the quadrotor appropriately performs normal maneuver and dead-end phase I and II maneuvers as well as collision avoidance. Note that Figure 11c shows the reasonable distance between the quadrotor and the object through the entire mapping process.

### 4.4. Discussion

From the above simulations, it is shown that the quadrotor with a proposed guidance algorithm manages to map the indoor structures. Velocity vector and yaw commands are fundamental elements for the quadrotor to map the object data points that are currently acquired. On the other hand, exploration completion logic determines whether the UAV should continue mapping or not, based on the accumulated object data points. During the discretization process to form the image plane, it is unnecessary to make an excessively dense image plane because the number of pixels directly affects the computational complexity to examine both exploration completion logic and dead-end situation logic. Due to the same reason, an interval between the trajectory of the quadrotor can be considered by a designer when conducting the proposed procedure with distance transform image plane. As the update rate of the guidance law depends on the sampling time of object data acquisition, adjusting the velocity magnitude command is important. Between the updates, the quadrotor can only maneuver with the latest updated information. In addition, if the velocity magnitude is not adequately adjusted, the quadrotor may dangerously encounter the object in the direction of movement ($+y^{b}$) before turning its yaw angle, as shown in Figure 6c and Figure 7c. When the vehicle enters the proposed dead-end phase I maneuver, the quadrotor immediately falls back with $v_{c}=v_{0}$. This helps to save time on the mapping procedure, compared to the previous algorithm with no dead-end situation logic.

Some issues are addressed for an experimental trial. The Indoor Positioning System (IPS) must be installed in the test environment because the position of the quadrotor is used in Equations (6), (8), (12), (13), and (17). As these equations directly affect the performance of the proposed guidance algorithm, accurate position information must be provided in real time. In addition, the quadrotor should be equipped with an Attitude and Heading Reference System (AHRS) for attitude information. Note that a depth sensing device should be carefully selected with the consideration of sampling time of data acquisition and resolution specifications. The embedded system should be mounted on the quadrotor to execute the proposed guidance algorithm while the object data information is fed from the depth sensing device. Then, the embedded system generates the angular velocity command for four rotors of the quadrotor. Vibration is indeed a big issue for the UAV in practice. Generally, to address this problem, a gimbal system can be considered to install the depth sensing device. In addition, vibration proof rubber may be used at every junction of the frame. Based on the parameters of the quadrotor including its control system and the performance of the depth sensing device, mapping parameters in Table 1 should be delicately designed. Trial and error approach may be inevitable during this procedure. Finally, since the proposed guidance algorithm must be computed in real time, a designer should figure out the elements that can reduce the computational load of the embedded system while not affecting the mapping performance seriously.

## 5. Conclusions

In this study, a guidance algorithm with a rotary-wing UAV for mapping unknown indoor structure is proposed. Object data points are collected by a sensor with a limited sensing range. The clustering method is conducted if multiple groups of points are detected at the same time. A velocity vector command is generated to track undiscovered area and maintain a predefined distance from the object. In addition, a yaw command is adjusted to be oriented toward the obtained object data points. The magnitude of the velocity command is modified to prevent a collision. A distance transform of a binary image formed with collected object data points is applied to establish exploration completion logic and dead-end situation logic. Straight lines starting from the current position should be examined to determine whether the quadrotor is in a dead-end situation or not. The discovered area should also be updated for the proposed logic. If a dead-end situation occurs, the guidance algorithm is switched to the proposed dead-end phase maneuver to escape from the current situation. Three numerical simulation results are shown to verify the proposed guidance algorithm. The results demonstrate that the quadrotor uses the proposed strategy to map various shapes of indoor environment without experiencing a collision. Dead-end situations are properly handled, compared to the previous algorithm. For future research, a flight test using an embedded system mounted on a quadrotor will be performed to validate the proposed guidance algorithm. In addition, the proposed algorithm will be extended to deal with a multi-floor structure. The vertical movement between two floors is additionally required for this case. In addition, the current algorithm can be handled by multiple UAVs, instead of one. An appropriate task assignment method must be considered to efficiently map indoor environments with multiple UAVs.

## Figures and Tables

**Table 1:** Parameters of the quadrotor and mapping used in numerical simulation.

*m*	6.2 kg	*d*	2.5 m	$\ell_{y_{max}}$	2.5 m
$J_{x}$	2.85 × 10${}^{-1}$ kgm${}^{2}$	$\ell_{id}$	1.5 m	$\ell_{y_{min}}$	2.2 m
$J_{y}$	2.85 × 10${}^{-1}$ kgm${}^{2}$	$c_{bf}$	0.35	$v_{0}$	0.8 m/s
$J_{z}$	4.94 × 10${}^{-1}$ kgm${}^{2}$	$c_{z}$	0.02	$v_{min}$	0.3 m/s
*g*	9.807 m/s${}^{2}$	$t_{m3}$	11.0 s	$\ell_{yd}$	0.15 m
